# Concurrent Gender Dysphoria/Incongruence and Autism Spectrum Disorder, a literature review

**DOI:** 10.1192/j.eurpsy.2024.995

**Published:** 2024-08-27

**Authors:** N. Clementi

**Affiliations:** ^1^General Adult Psychiatry/ Gender Identity Clinic, Royal Cornhill Hospital, NHS Grampian; ^2^University of Aberdeen, School of Medicine, Aberdeen, United Kingdom

## Abstract

**Introduction:**

Several studies have found that ASD (Autism Spectrum Disorder) and GD (Gender Dysphoria by DSM-V)/GI (Gender Incongruence by ICD-11) tend to co-occur, and in recent years the interest and publications on this comorbidity has increased rapidly.

**Objectives:**

To review the prevalence of ASD in individual with a diagnosis of GD/GI.

To better tailor and improve care offered in the National Health Service (NHS) Gender Identity Clinics (GICs) throughout the UK.

**Methods:**

Systematic literature review was conducted via Pub Med, MEDLINE and PsycINFO by the author, for all English-language articles published between 2018 and 2023, containing keywords as ASD, GD (Gender Dysphoria), GI (Gender Incongruence), transgender, autistic traits, autism, gender diversity, gender variance.

**Results:**

Rate of people with ASD appear to be higher in people accessing Gender Identity Clinics (GICs) than in the general population. Results from this literature review show increased prevalence of GD and GI in ASD population.

**Conclusions:**

This comorbidity has highlighted the importance of better tailor transgender healthcare services for people with neurodevelopmental conditions and neurodiversity, to avoid delay in ASD individuals accessing care and gender affirming medical treatments. Services should strive to provide an effective and equitable service. It is also important to better identify potential barriers for ASD people in accessing gender care. Literature also shows the people with ASD have more difficulties in communicating and describing their gender narrative and to express their wishes for gender treatments. Symptoms including problems in communications and social skills, obsession and rigidity can also impact their assessment of GD/GI in gender identity services. Some studies showed that for individuals who have concurrent ASD and GD/GI, assessment in GICs may be extended to better review their wishes for gender identity and for gender affirming treatment. Further research is needed to better investigate and understand factors explaining the relationship between ASD and gender diversity. There is still limited research in the real life experiences of gender diverse and autistic people. There is also a need to improve Gender reassignment protocol nationally to better care for individual with ASD and GD/GI throughout GICs in the UK.

**Disclosure of Interest:**

None Declared

